# Recurrence characteristics and clinicopathological results of borderline ovarian tumors

**DOI:** 10.1186/s12905-021-01263-y

**Published:** 2021-03-31

**Authors:** Lina Niu, Huihui Tian, Yongjun Xu, Jieqiong Cao, Xu Zhang, Junli Zhang, Jiajia Hou, Weiqin Lv, Junxia Wang, Li Xin, XuFeng Dong, Tao Xu, Yuan Nan, Hua Wei, Xinting Chai, Na Li, Yan Ni, Yun Shang, Lizhen Zhang, Ye Zhao

**Affiliations:** 1 Department of Gynecology, Yuncheng Central Hospital of Shanxi Province, Yuncheng, 044000 China; 2 Department of Pharmacy, Yuncheng Central Hospital of Shanxi Province, Yuncheng, 044000 China; 3 Department of Gynecology, First Hospital of Shanxi Medical University, NO.85 South Jiefang Road, Yingze District, Taiyuan, 030001 China; 4 Department of Infectious Disease Prevention and Control Division, Shanxi Center for Disease Control and Prevention, Taiyuan, 030012 China; 5 Department of Pathology, Yuncheng Central Hospital of Shanxi Province, Yuncheng, 044000 China; 6 Department of Medical Record Management, Yuncheng Central Hospital of Shanxi Province, Yuncheng, 044000 China; 7 Department of Gynecology, Taiyuan Central Hospital, Taiyuan, 030000 China

**Keywords:** Ovarian borderline tumor, Recurrence, FIGO staging, Conservative surgery, CA125

## Abstract

**Background:**

This study aimed to investigate the clinical and pathological characteristics, and the recurrence and prognostic factors of borderline ovarian tumors (BOTs).

**Methods:**

The data of 286 patients admitted to hospital and followed up for more than ten months were analyzed retrospectively to study the clinicopathological characteristics and related factors of recurrence.

**Results:**

The median age of the patients was 42.06 ± 14.97 years, and the duration of the follow-up ranged from 10–109 months. During the follow-up period, 40 patients had a recurrence. Of these patients, 36 were ≤ 40 years, and patients with premenopausal recurrence accounted for 20.5% (36/176). In patients undergoing conservative treatment or radical operations, the recurrence rates were 21.3% and 1.8%, respectively, and they were 13.4% (36/268) in patients at Federation International of Gynecology and Obstetrics (FIGO) stage I, and 22.2% (4/18) in patients at an advanced stage. Postoperative pathology revealed that 40 patients had micropapillary tumors, among whom ten patients (25%) had a recurrence, and 19 patients had complications with interstitial infiltration. Of these 19 patients, six had a recurrence (31.5%). Another 22 patients had complications with calcified sand bodies; among these, eight patients (36.4%) had a recurrence. All the differences were statistically significant (*P* < 0.05). There were four cancer-related deaths during the follow-up period. Late FIGO stage, conservative operation, and a high level of carbohydrate antigen 125 (CA125) were independent risk factors for the recurrence of BOTs.

**Conclusion:**

BOTs usually occur in women under 40 years, have an occult onset, and half of the patients have no obvious clinical manifestations. Serum CA125 level can be used as a tumor marker to detect BOTs and the risk of its recurrence. Operation mode and FIGO stage are important independent factors for the recurrence of BOTs.

## Background

Borderline ovarian tumors (BOTs) are ovarian tumors with growth patterns and cytological characteristics that fall between benign and malignant tumors, and non-destructive stromal infiltration. Prognosis is much better than that of ovarian cancer of the same clinical stage [1, 4]. BOTs account for 10%–15% of ovarian epithelial tumors, and their staging is based on the FIGO staging system of ovarian cancer [2]. At the time of diagnosis, 75% of patients with BOTs are at an early stage. Only a few patients are in the late stage at diagnosis [3]. Plasma mucinous tumors are mucinous cystadenoma that accounts for 20% of benign ovarian neoplasms. 95% of the tumors are unilateral, grayish-white, large or large, often multilocular in section, with varying lumen sizes. Cyst septum is composed of connective tissue, cyst fluid is jelly-like, containing mucin or glycoprotein, tumor surface is smooth, there is little nipple growth, cyst cavity is covered by a single layer of tall columnar epithelium, can produce mucus. The malignant change rate is 5–10%.

The standard treatment for BOTs is surgery, but there is no standard surgical procedure [4]. Operations are generally conservative, i.e., the removal of the adnexa on the affected side. The pathological results determine whether it is necessary to carry out an ovarian cancer re-staging operation or chemotherapy. Chemotherapy is not recommended for borderline tumors if there are no invasive tumors or implants but it is suitable for young patients with fertility requirements. The radical operation, on the other hand, is a total hysterectomy or bilateral adnexectomy and is, thus, more suitable for patients who no longer wish to have children [4].

Most BOTs can be diagnosed in the early stage, but it is reported that the recurrence rate ranges from 5 to 20% [5], and the recurrence and mortality rates have gradually increased. While the BOT onset age has dropped in recent years, with improvements in the quality of life and the implementation of the two-child policy in China, more and more patients wish to retain their reproductive function. Therefore, determining the clinicopathological characteristics of patients who are prone to relapse, selecting patients needing fertility-preserving surgery, devising a follow-up plan, and determining whether to carry out the second comprehensive staging surgery have become issues of concern to our clinicians. This study retrospectively analyzed the clinical and pathological data of patients with BOTs and identified the risk factors related to the recurrence of BOTs. It is of great significance for the diagnosis, treatment, and follow-up management of patients with BOTs.

This study collected and analyzed the clinicopathological and follow-up data of 286 patients with BOT, admitted to two hospitals between 2010 and 2018. The investigators studied all the clinical and histopathological factors that may be related to recurrence and obtained new data related to it.

## Methods

### Patients

The data of 286 patients with ovarian masses, who were admitted to and operated on in the First Affiliated Hospital of Shanxi Medical University and Yuncheng Central Hospital between January 2010 and December 2018, were analyzed retrospectively.

The inclusion criteria were as follows: (1) patients with BOTs that were confirmed by pathology; (2) patients with complete clinical-pathological data and whose diagnoses were based on the World Health Organization (WHO) pathological diagnosis standard of BOTs; and, (3) patients with complete follow-up data.

The exclusion criteria were as follows: (1) patients whose intraoperative frozen sample was borderline, but postoperative pathology was malignant or benign; (2) patients with other tumor histories or without key medical records or key follow-up information; and, (3) patients with serious cardiovascular and cerebrovascular accidents. Among the 286 patients, 40 patients with recurrence were assigned as the recurrence group, and the remaining 246 without recurrence were assigned as the control group.

The median age of the patients was 42.06 ± 14.97 years, so the age factor was set to be > 40 and ≤ 40.

### Data collection and observation indicators

The clinical case data of this study were all from the medical record data. The clinical-pathological data concerned age, menstruation cycle, clinical symptoms, B-ultrasound results (tumor maximum diameter line), CA125, surgical approach and type, FIGO stages, and postoperative pathology.

Two senior pathologists from both hospitals reviewed the pathological results of the recurrence group. Follow-up data came from telephone inquiries and outpatient re-examinations. The deadline for follow-up was September 30, 2019. The follow-up time of patients with BOTs was 10–109 months, and follow-up only ended in cases of death. The postoperative recurrence of BOTs refers to the emergence of a tumor with the same biological type as the original tumor after complete resection [6].

### Statistical analysis

Data were statistically analyzed using SPSS 22.0 statistical software. Measurement data were evaluated using a t-test or analysis of variance, and count data were evaluated using the χ^2^ test or Fisher exact probability test, α = 0.05. A logistic regression model was used to analyze the variables with a *P* value < 0.10 in univariate analysis.

## Results

### Analysis of the factors influencing the recurrence of BOTs

Tables 1 and 2 contain the data of patients with BOTs included in the study. It was found that the recurrence rate of BOTs was 13.9% (40/286). The differences in age, menstrual cycle, B-ultrasound result (tumor size), microinvasion, micro papilla, calcified sand bodies, the FIGO stage, and operation type between the recurrence group and control group were statistically significant (*P* < 0.05).

**Table 1:** Analysis of clinical features and influencing factors of the two groups [n(%)]

	Factor	N (%)	Recurrence		χ^2^	*P*
			Yes	No		
Age stratification	≤ 40	136(47.6)	36	100	33.597	< 0.001
	> 40	150(52.4)	4	146		
Menstrual condition	Menorrhea	176 (61.5)	31	145	7.766	0.021
	Menopause	74 (25.9)	5	69		
	Other	36 (12.6)	4	32		
Clinical symptoms	Asymptomatic masses	176 (62.9)				
	Pain in the lower abdomen	40 (14.3)				
	Abdominal distention and discomfort	34 (12.1)				
	Abnormal uterine bleeding	20 (7.1)				
	Urinary frequency and urgent urination	10 (3.6)				
B-ultrasound result (tumor size)	≤ 10 cm	144 (50.4)	27	117	5.786	0.022
	> 10 cm	142 (49.6)	13	129		
CA125	Normal	114 (44.5)	10	104	3.628	0.065
	Rise	142 (55.5)	24	118

**Table 2:** Chi-square analysis of surgical pathological features and recurrence of the patients [n(%)]

Factor	N (%)	Recurrence		χ^2^	*P*
FIGO stage					
Stage I	268 (93.7)	36	232	20.281	< 0.001
Stage II	6 (2.1)	2	4		
Stage III	12 (4.2)	2	10		
Micro papilla					
Yes	40 (14.0)	10	30	4.689	0.046
No	246 (86.0)	30	216		
Microinvasion					
Yes	19 (6.6)	6	13	5.236	0.034
No	267 (93.4)	34	233		
Focal cancerous					
Yes	58 (21.3)	4	54	2.116	0.182
No	228 (78.7)	30	198		
Calcified sand bodies					
Yes	22 (7.7)	8	14	15.274	0.01
No	264 (2.3)	22	244		
Surgical approach					
Laparotomy	180	28	152	0.403	0.585
Laparoscope	106	12	94		
The operation type					
Conservative operation	178 (61.7)	38	140	16.894	< 0.001
Whether the affected appendage should be excised	83 (28.7)	15	68		0.212 (exact probability)
Resection of lymph					
Whether or not the affected side of the ovarian cyst stripping	71 (25.2)	15	56	2.971	0.085
Radical operation	108 (38.3)	2	106		
Yes	40	2	38		
No	245	37	208		
	286				
Serous	126 (44.1)	16	110	3.711	0.262
Histological type					
Mucinous tumors	138 (48.3)	20	118		
Plasma mucinous tumors	12 (4.2)	4	8		
Endometriosis	4 (1.4)	0	4		
Brenner	2 (0.7)	0	0		
Clear cell tumor	4 (1.4)	0	0		

Clinical symptoms: 176 patients (62.9%) had asymptomatic masses, 40 patients (14.3%) had pain in the lower abdomen, 34 patients (12.1%) had abdominal distention and discomfort, 20 patients (7.1%) had abnormal uterine bleeding, and ten patients (3.6%) had urinary frequency and urgent urination.

### Analysis of the factors influencing the recurrence of BOTs

Table 2 shows the relevant parameters of logistic multivariate regression analysis for the possible risk factors of recurrence. The results revealed that the FIGO stage, the type of operation, and serum CA125 were independent risk factors for the recurrence of BOTs (Table 3).

**Table 3:** Logistic regression analysis of related factors of borderline ovarian tumor recurrence

	B	Wals χ^2^	*P*	Exp (B)	95% credibility interval
FIGO stage	2.303	3.927	0.048	10.000	1.026–21.458
Operation type					
Radical operation	2.627	12.671		1	
Conservative operation				13.826	3.256–25.719
CA125 normal or not					
Yes	1.013	4.444	0.043	1	1
Rise				2.755	1.132–7.458

Compared with the non-recurrence group, the higher the FIGO stage, the greater the risk of recurrence. The recurrence rate after having a conservative operation was 13.826 times that after a radical operation, and the recurrence rate of patients with increased CA125 was 2.755 times that of patients with normal CA125.

### Analysis of the survival curves

**Fig. 1:**
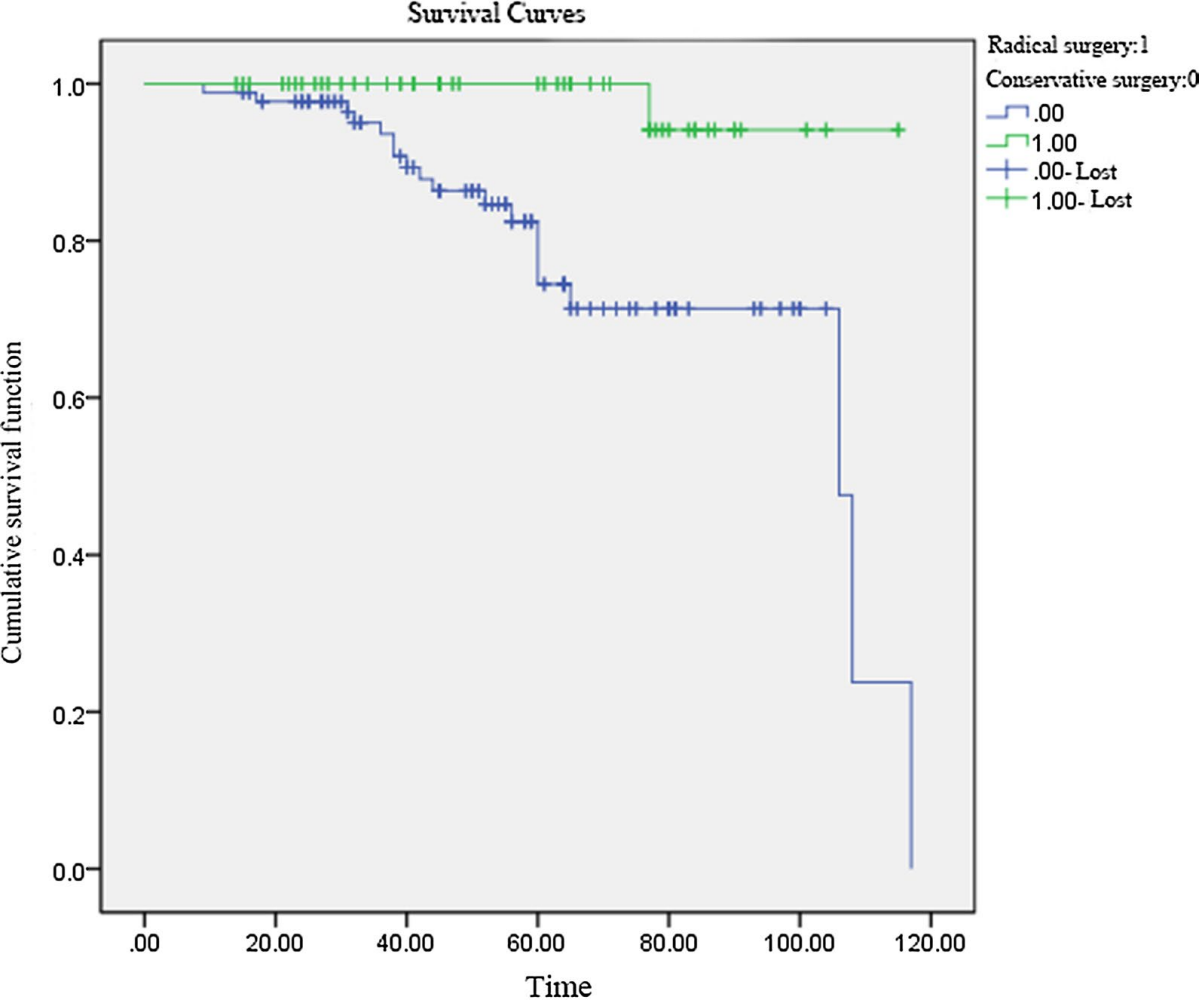
Difference in survival curves between the two groups

A univariate analysis of the follow-up results and recurrence indicated that the median follow-up time of these 286 patients with borderline tumors was 54 months (9–117 months), and 40 patients had a recurrence, the recurrence rate being 13.9%, and the median recurrence time being 48 months. Of the 178 patients who received conservative surgery, 38 had a recurrence (21.3%) and the median recurrence time was 44 months. Of the 108 patients undergoing radical surgery, there were two patients with a recurrence. The recurrence rate was 1.9%, and the median recurrence time was 77 months. Log-rank tests showed that the difference in survival curves between the two groups was statistically significant (*P* < 0.05) (see Fig. 1).

## Discussion

BOTS are tumors with low malignancy potential. It has been proven that although they have a good prognosis, they also have a notable recurrence rate. In this study, 286 patients with BOTs were followed up for 10–109 months, and pathology revealed that BOTs recurred in 40 patients, which gives a recurrence rate of 13.9%. Qian Zhang et al. [7] reported a lower recurrence rate of 9.2% but the recurrence rate in this study is consistent with other research. BOTs are mostly found in women of childbearing age and often only found during a physical examination due to the absence of clinical symptoms in the early stages. The present study revealed that the median age of the onset of BOTs was 42.06 ± 14.97 years. Asymptomatic pelvic masses were found in 62.9% of the asymptomatic patients through physical examination, while abnormal uterine bleeding, urinary frequency, and urgent urination occurred in only 7.1% and 3.6% of patients. The number of patients who experienced pain in the lower abdomen and abdominal distension and discomfort was slightly higher. However, these symptoms are not characteristic symptoms of BOTs, so they are easily overlooked. Annual physical examinations are clearly important.

The treatment of BOTs is based on the National Comprehensive Cancer Network (NCCN) guidelines, and gives thorough consideration to the characteristics of the disease, as well as the patient’s age and her desire to remain fertile [8]. There are two possible types of operation. The first is a conservative operation, which retains at least part of the uterus and unilateral ovary. It is suitable for younger patients with reproductive or endocrine requirements. The operation methods include unilateral tumor removal, bilateral tumor removal, unilateral adnexectomy, and unilateral adnexectomy + contralateral focus resection, and comprehensive staging of fertility preservation [9]. The second is a radical operation, which means, at the least, a hysterectomy and bilateral adnexectomy, omentectomy, and peritoneal washings/biopsies, including a comprehensive staging of ovarian cancer [9]. Borderline tumors have a good prognosis and a low recurrence rate, and if they do recur, they mostly remain borderline. That is to say, malignant transformation is rare, but there is still a risk of recurrence, disease progression, and death. Recurrence almost always occurs in the reserved ovary; therefore, the management of BOTs is controversial [10]. The present study has found that the recurrence rate in a conservative operation is higher than that of a radical operation (21.3% vs. 1.8%, *P* < 0.001), and multivariate analysis results indicate that conservative surgery is an independent risk factor for the recurrence of BOTs. As conservative surgery only removes the areas affected by the tumor, it is easy to ignore the seemingly unaffected parts. If the tumor is potentially cancerous, or cancer has occurred, the tumors are likely to have invaded other parts of the reproductive system [11]. However, for patients with fertility requirements, especially young patients, the radical operation will greatly affect their quality of life. One study revealed that not only does postoperative chemotherapy not necessarily improve patients' prognosis, but it can also lead to complications and increase the mortality rate. Therefore, chemotherapy is generally not recommended [11, 12]. The investigators believe that it is necessary to be cautious about only operating on patients conservatively, and stress that follow-up after the operation is essential, and a radical operation should be considered as early as possible after the birth of a child.

In a previous study, postoperative pathology revealed that the recurrence rate was higher in patients with micropapillary and microinvasion tumors [13], which have been proven to be related to a variety of malignant tumors, ovarian cancer being one of them [14]. One scholar considers that when the composition of a micropapillary tumor is less than 25% or less than 10%, the severity of the lymphatic invasion and lymph node metastasis is also significantly higher than that of patients without micropapillary tumors. Therefore, as long as the tumor has micropapillary components, it should be diagnosed as invasive micropapillary carcinoma [14], and this is why clinicians should pay attention to micropapillary and microinvasion tumors. Calcified psammoma bodies refer to calcified bodies with concentric circles, and calcified psammoma bodies can be detected in serous cystadenocarcinoma and serous mucinous carcinoma of the ovary [15]. Whether calcified psammoma bodies are related to recurrence is still a controversial theory. One study concluded that the formation of calcified sand bodies, which need to be formed under the condition of better cell differentiation, is slow. This indicates that the biological behavior of a tumor that can form calcified psammoma bodies is an improvement. Therefore, calcified psammoma bodies may also be an indicator of good tumor biological behavior [16]. However, the present study has revealed that micropapillary, microinvasion, and calcification of psammoma bodies in patients were related to recurrence. It suggests that short-term and long-term follow-up are extremely important for patients with pathologically confirmed micropapillary, microinvasion, and calcified sand bodies, and especially for those who have undergone conservative surgery. It is recommended that a radical operation should be performed as early as possible for patients who have had a conservative operation and who have completed their reproductive plans.

Almost all recurrences occur in the pelvic cavity, and recurrences outside the ovary are rare. Furthermore, patients with BOTs at the late FIGO stage are prone to experience recurrence. A study revealed that FIGO staging was an independent risk factor for the recurrence of BOTs. The higher the FIGO stage, the greater the potential of tumor recurrence [17]. This is consistent with the results of this study. CA125 is a commonly used clinical marker for monitoring ovarian cancer and is of great significance in diagnosing and treating ovarian cancer. However, it remains controversial whether it is related to the recurrence of borderline tumors [18]. The present study demonstrated that CA125 was related to it. Indeed, further multivariate analysis showed that CA125 is an independent risk factor for the recurrence of BOTs, and the risk of recurrence was significantly higher in patients with elevated CA125. Therefore, the investigators used the CA125 level as a screening and postoperative re-examination monitoring index for patients with BOTs, and paid special attention to patients with elevated CA125 levels.

Despite being more detailed and comprehensive in terms of data than previous studies, there are some limitations to this study. Although the medical records were well preserved, and the case records such as height, weight, menstruation cycle, body mass index, and pathological data were detailed, because this study was retrospective, some data were still unavailable. Second, although the sample size in this study was not small, the larger it is, the more reliable the findings are. Moreover, the samples in this study were collected from two hospitals, and the levels of examination, operation, intraoperative freezing, and postoperative pathological tests of the medical professionals in the different hospitals may have been affected by this to some extent. In addition, the recurrence time of this disease is mostly within 10–20 years, so the short follow-up time of patients may also have influenced the analysis results in this study.

In summary, this disease has an early onset, a good prognosis, and a low recurrence rate, and tumors are mostly borderline after recurrence. Since patients can survive for a long time, it is desirable to retain fertility function. However, because it has a certain recurrence rate, canceration rate, and mortality rate, the investigators recommend that patients with any of the above recurrence risk factors should be followed up for a long time [8]. Patients without follow-up opportunities or patients who have undergone conservative surgery and completed their reproductive plans should consider undergoing radical surgery to avoid any recurrence and the possibility of death.

## Conclusion

The present study reveals that BOTs usually occur in young women (under 40 years) and have an occult onset; many patients, in this study half of them, have no obvious clinical manifestations. Serum CA125 levels can be used as a tumor marker to detect BOTs and the risk of their recurrence. In addition, operation modes and the FIGO stage are important independent factors for the recurrence of BOTs.

## Data Availability

The data generated during the current study will be available from the corresponding author on reasonable request and in accordance with the consent and ethical approval.

## Abbreviations

BOTs: borderline ovarian tumors
FIGO: Federation International of Gynecology and Obstetrics
WHO: World Health Organization
NCCN: National Comprehensive Cancer Network
